# Dietary Supplementation with *Flammulina velutipes* Stem Waste on Growth Performance, Fecal Short Chain Fatty Acids and Serum Profile in Weaned Piglets

**DOI:** 10.3390/ani10010082

**Published:** 2020-01-03

**Authors:** Xuzhou Liu, Jinbiao Zhao, Gang Zhang, Jiangxu Hu, Ling Liu, Xiangshu Piao, Shuai Zhang, Yu Li

**Affiliations:** 1 Institute of Mycology, Engineering Research Center of Chinese Ministry of Education for Edible and Medicinal Fungi, Jilin Agricultural University, Changchun 130118, China; 15204400015@139.com; 2 State Key Laboratory of Animal Nutrition, Ministry of Agriculture Feed Industry Centre, China Agricultural University, Beijing 100193, China; 15600911358@163.com (J.Z.); 18729564631@163.com (G.Z.); hujx1007@163.com (J.H.); 18435132241@163.com (L.L.); piaoxshu@cau.edu.cn (X.P.)

**Keywords:** *Flammulina velutipes* stem waste, growth performance, short chain fatty acid, weaned piglets

## Abstract

**Simple Summary:**

*Flammulina velutipes* stem waste (FVS) is the by-product of *Flammulina velutipes* (FV), which is rich in amino acids, vitamins and trace minerals. The direct disposal of FVS can cause serious environmental pollution. Therefore, the objective of this study was to determine the utilization and effects of FVS in diets for weaned piglets. Effective utilization of FVS can avoid the waste of resources, and have direct positive effects on environmental pollution reduction.

**Abstract:**

This study was conducted to evaluate the effects of dietary FVS supplementation on growth performance, nutrient digestibility, biochemical profile of serum and fecal short chain fatty acids (SCFAs) production in weaned piglets. In Exp.1, 150 weaned pigs (initial body weight: 6.89 ± 1.17 kg) were allotted to five dietary treatments. The treatment diets included a basal diet and four experimental diets supplemented with 2.5%, 5.0%, 7.5% and 10.0% FVS respectively. The animal trial lasted for 28 days. In Exp.2, 72 piglets (initial body weight: 8.20 ± 1.67 kg) were allotted to three dietary treatments. The treatment diets included a basal diet and two experimental diets supplemented with 1.5% and 3.0% FVS, respectively. The animal trial lasted for 56 days. The results showed that pigs fed dietary FVS with 3% or lower inclusion levels had no significant difference (*p* > 0.10) on growth performance compared with pigs fed the control diet during day 1–28 and day 1–56. Dietary FVS supplementation decreased the apparent total tract digestibility (ATTD) of nutrients on day 28, day 35 and day 56, but no significant changes (*p* > 0.05) of nutrient digestibility were observed on day 14. Although piglets fed diets with higher levels of FVS showed impaired growth performance and ATTD of nutrients, dietary FVS supplementation improved the fecal SCFA production, antioxidant capacity, interleukin-2 and growth hormone levels in serum, and reduced the harmful low-density lipoprotein levels in serum on day 56. In conclusion, as a promising alternative fibrous ingredient, FVS could be supplemented in diets of weaned piglets with a proportion under 3%.

## 1. Introduction

Mushrooms are excellent sources for selenium, vitamin B such as riboflavin, and minerals such as potassium and copper, and are also rich in dietary fiber (DF), β-glucans and chitin [1]. *Flammulina velutipes* (FV) is the fourth most popular edible mushroom worldwide because of its delicious taste and high nutritional properties, which contains high proportion of essential amino acids, vitamins, and fiber, low energy and fat content [2]. Particularly, FV occupies large proportion in the edible mushroom market in Asia. With the continuous expansion of the FV production, the by-product of FV, *Flammulina velutipes* stem waste (FVS), has also been produced in large amounts. Due to its high moisture and nutrient content, FVS could become spoilage easily, and has a pungent odor. Although some companies were trying to produce biological fertilizer using FVS, most FVS was mixed into coal and burned in a boiler. Therefore, FVS has not been properly utilized so far. The common management of FVS was a waste of resources, and the direct disposal of FVS may cause environmental pollution.

Dietary fiber plays an important role in piglet feeding and has received increasing attention in recent years. A minimum level of DF has to be supplemented in diets to maintain the normal physiological function of weaned piglets [3]. Although some kinds of DFs are associated with impaired nutrient digestibility and low energy value, dietary DF supplementation also possess many positive effects, such as improving the satiety, stimulating gut health and enhancing animal well-being. The effects of DF on piglet health and nutrition depend on the fiber properties and may differ greatly between fiber sources [4]. Schiavon et al. (2004) [5] reported that 12% beet pulp supplementation in diets of weaned piglets did not affect the feed intake and feed conversion ratio. Molist et al. (2009) [6] reported that piglets consumed wheat bran showed elevated feed intake compared with those diets without dietary fiber containing, and they also observed that increased content of non-starch polysaccharides in diets may improve the abundance of beneficial microbiota in the gut.

In recent years, some studies reported that dietary FVS supplementation in broilers had a beneficial effect on growth performance, increased the short chain fatty acids (SCFAs) production in the intestine [7], and improved the villus height to crypt depth ratio, interleukin-2 (IL-2), interleukin-4 (IL-4) and S-immunoglobulin A (S-IgA) contents in the intestine of broilers [8,9]. To our knowledge, there are few studies on the utilization of FVS in pigs. Chu et al. (2012) [10] reported that diets supplemented with fermented FV by-product decreased the growth performance, but improved the carcass grade when fed to growing-fattening pigs. However, there is still a lack of basic data for the utilization of FVS as a feed ingredient in diets of weaned piglets.

Therefore, the objective of this study was to determine the effects of dietary FVS supplementation at different inclusion levels on growth performance, apparent total tract digestibility (ATTD) of nutrients, biochemical profiles in serum and fecal SCFAs production in weaned pigs, and to test whether the FVS could be used as a feed ingredient fed to weaned pigs as to broilers.

## 2. Materials and Methods

All procedures used in these experiments received prior approval from the Institutional Animal Care and Use Committee of China Agricultural University (ID: SKLABB-2010-003).

The animal trials were conducted in the Swine Nutrition Research Center of the National Feed Engineering Technology Research Center (Chengde, China). The FVS was provided by Changchun Ficus Altissima Biotechnology Company (Changchun, China), and was processed in Jilin Green Biological Technology Company (Siping, China). Specifically, the fresh FVS was dried at 65 °C using a triple-pass rotary drum dryer machine (Jiutai Rotary dryer, Jiangsu, China) and then ground in a hammer mill using a 2 mm screen into powder before including into the diets. The same batch of FVS was used for all experiments. The analyzed chemical compositions of FVS were shown in Table 1.

### 2.1. Animal, Diets, Experimental Design and Sample Collection

The digestible energy (DE) value (4.11 MJ/kg as fed basis) and the standardized ileal digestible (SID) amino acid values (ranged from 17.50% to 59.47%) of FVS fed to pigs had been determined using the total feces collection method and the difference method prior to this study (data not shown), and all the diets used in the current study were formulated based on these values.

In Exp.1, 150 weaned piglets (initial body weight (BW): 6.89 ± 1.17 kg, weaned at 28 days of age) were assigned to five treatments in a completely randomized design. Each treatment diet was fed to six replicated pens, with five replicated pigs (three barrows and two gilts or two barrows and three gilts, balanced between treatments) per pen. The treatment diets included a control diet and four experimental diets formulated by supplementing with 2.5%, 5%, 7.5% or 10% FVS, respectively (Table 2). The ratios of DE to crude protein (CP), SID Lys to DE, SID Lys to SID Met, SID Lys to SID Thr and SID Lys to SID Trp in all five diets were kept the same or close. No antibiotics or any other growth promoters were added to the five diets during the animal trial. All diets were supplied with 0.3% chromic oxide as an indigestible marker in the last 2 weeks.

All pigs were housed in pens with drinkers, feeders and slatted floors, and were given free access to feed and water. The environment temperature of the barn was controlled between 25–30 °C, and the relative humidity was controlled at 55–65%. The animal trial lasted for 28 days. Pigs and feed were weighed at the beginning of the experiment (d 1), mid-stage (d 14) and the end (d 28) to determine the average daily gain (ADG), average daily feed intake (ADFI) and gain-to-feed ratio (G/F). Diarrhea rate was determined according to the procedures described by Yu et al. [11]. Briefly, pigs were observed and recorded daily for clinical signs of diarrhea, and diarrhea rate was calculated on pen basis as the proportion of days in which piglets showed clinical signs of diarrhea with respect to the total number of trial days. The number of deaths in each pen was recorded for different phases, and the mortality ratio was expressed as a percentage of total piglets according to the method by Feyera et al. [12]. The fecal and blood samples were handled as described by Zhao et al. [13]. From d 26–28, approximately 100 g of fresh feces were collected from each pen for 3 days, and were stored at −20 °C. All fecal samples were pooled by pen and then dried at 65 °C for 72 h. The fresh feces were collected directly from a rectum on d 14 and d 28, and immediately analyzed for SCFAs concentrations. Blood samples were collected by precaval vein puncture into 10 mL vacuette tubes on d 28. All the samples were collected from pigs with BW close to the average BW of each pen. Serum samples were gained from blood samples collected after centrifugation at 3000 r/s for 15 min and stored at −20 °C until analysis.

Exp.2 was designed based on the results of Exp.1, and the inclusion level of FVS in experimental diets was decreased to 1.5% or 3%. Longer adaptation time was expected for pigs to digest dietary fiber [14], so we expended the animal trial to 8 weeks, including three phases (phase 1: from d 1 to d 14, with BW of 7–11 kg; phase 2: from d 15 to d 35, with BW of 11–25 kg; phase 3: from d 36 to d 56, with BW of 25–50 kg). The diets in each phase were formulated to keep the same DE, SID Lys, SID Met, SID Thr and SID Trp levels, and to meet the nutrient requirements recommended by NRC (2012) [15]. A total of 72 healthy weaned piglets (initial BW: 8.20 ± 1.67 kg, weaned at 28 days of age) were assigned to three treatments in a randomized complete design. Each treatment diet was fed to six replicate pens, with four replicated piglets (two barrows and two gilts) per pen. The treatment diets included a control diet and two experimental diets formulated by supplementing with 1.5% and 3% FVS, respectively (Table 3). All pigs were housed in pens with slatted floors and had free access to feed and water throughout the 56-d experiment. The temperature inside the barn was controlled between 25 and 30 °C, and the relative humidity inside the barn was controlled at 55–65%. Pigs and feed were weighed at the beginning (d 0), the end of phase 1 (d 14), phase 2 (d 35) and phase 3 (d 56) to determine the ADG, ADFI and G/F. Diarrhea rate and mortality ratio were determined using the same procedure as in Exp.1. The fecal and blood samples were collected and processed using the same procedures as in Exp.1.

### 2.2. Chemical Analyses

All ingredients, diets and feces samples in Exp.1 and 2 were ground to pass through a 1 mm screen, mixed thoroughly and assayed in duplicate. The FVS, diets and feces samples in Exp.1 and 2 were analyzed for gross energy (GE) using an Automatic Isoperibol Oxygen Bomb Calorimeter with benzoic acid as calibrator (model 6300, Parr Instruments, Moline, IL, USA). Moreover, the FVS, diets and feces in Exp.1 and 2 were analyzed for dry matter (DM, procedure 930.15; AOAC International, 2006) [16], CP (procedure 984.13; AOAC International, 2006), ash (procedure 942.05; AOAC International, 2006), ether extract (EE, procedure 920.39; AOAC International, 2006), calcium (procedure 968.08; AOAC International, 2006), phosphors (procedure 946.06; AOAC International, 2006), neutral detergent fiber (NDF) and acid detergent fiber (ADF) [17]. The NDF and ADF contents were determined using fiber bags and fiber analyzer equipment (model 200, ANKOM Technology Corp., Macedon, NY, USA). The NDF was analyzed with heat-stable α-amylase and sodium sulfate without correction for insoluble ash. The ADF fraction was expressed with the inclusion of crude ash. Lignin was detected as reported by Dence [18]. The content of chromium in diets and feces was measured using an Atomic Absorption Spectrophotometer (model Z-5000, Hitachi Corp., Tokyo, Japan) according to the method of Williams et al. [19].

The fresh fecal samples were thawed at 4 °C and processed as described by Wu et al. [20]. The concentrations of SCFAs were determined via ion chromatography (model ICS 3000, Thermo Crop., Sunnyvale, CA). The concentrations of serumal immunoglobulins (IgA, IgG and IgM) were measured by an ELISA test kit (IgA, IgG and IgM quantitation kit; Beijing Sino-UK Institute of Biological Technology, Beijing, China). The growth hormone (GH) was measured following the protocol of Strasburger [21]. Antioxidant parameters including glutathione peroxidase (GSH-Px), superoxide dismutase (SOD), catalase (CAT), total antioxidant capacity (T-AOC) and malondialdehyde (MDA) were determined using assay kits following the manufacturer’s instructions (Beijing Sino-UK Institute of Biological Technology, Beijing, China). Interleukin-2 (IL-2) and interleukin-6 (IL-6) were determined using commercially available porcine ELISA kits (Beijing Sino-UK Institute of Biological Technology, Beijing, China). The contents of urea, high density lipoprotein (HDL), low density lipoprotein (LDL), albumin (ALB), total protein (TP), total cholesterol (TC), triglyceride (TG) and glucose (GLU) in serum were determined using automatic biochemical analyzer (model 7170, Hitachi Corp., Tokyo, Japan) with corresponding kits (BioSino Bio-Technology and Science Inc., Beijing, China).

### 2.3. Calculation

The ATTD of nutrients (DM, OM, GE, CP, EE, NDF and ADF) were calculated using equations described by Kong et al. [22]: Digestibility (%) = 100 − [(CI_input_ × CC_output_/CI_output_ × CC_input_) × 100], in which CI_input_ and CI_output_ are the concentration of chromic oxide in diets and feces, respectively, and CC_input_ and CC_output_ are the concentrations of nutrients in diets and feces, respectively.

### 2.4. Statistical Analysis

Data of growth performance, nutrient digestibility, biochemical indices in serum and SCFA concentrations gained in Exp.1 and Exp.2 were checked for normality and outliers using the PROC UNIVARIATE procedure of SAS 9.2 (SAS Inst. Inc., Cary, NC, USA). Outliers were identified using cook’s distance and abandoned when analyzing data. Then data were analyzed using the PROC GLM procedure of SAS. The treatment diet was the only fixed effect and the pen was treated as the experimental unit. The LSMEANS statement was used to separate treatment means, with Turkey’s test for adjustment. Moreover, orthogonal polynomial contrasts were conducted to determine the linear and quadratic responses of the increased dietary FVS levels on other parameters. Furthermore, rates of mortality in each stage in both experiments were analyzed using a logistic regression (GENMOD procedure), using a binomial error distribution. The link function was a logit-transformation. Results of the logistic regression were then converted back to (original) natural units. Significant differences were declared at *p* < 0.05.

## 3. Results

### 3.1. Exp.1

The ADG and ADFI significantly decreased for piglets fed diets with 5% and higher levels of FVS compared to those fed the control diet and diet with 2.5% FVS in the first 2 weeks (*p* < 0.01) (Table 4). The ADG and ADFI linearly decreased (*p* < 0.01) with increased inclusion levels of FVS during d 1 to 14. However, there was no difference in G/F during this period. Moreover, no differences were observed in ADG, ADFI and G/F during d 15 to d 28, but the ADG and G/F linearly decreased (*p* < 0.01) with increased FVS levels during this period. The ADG and G/F also significantly decreased for piglets fed diets with 5% and higher levels of FVS compared to those fed the control diet and diet with 2.5% FVS during d 1 to d 28 (*p* ≤ 0.01). The ADG and G/F linearly decreased (*p* < 0.01) with increased FVS levels during the whole experiment. There were no differences on diarrhea rate and mortality ratio among the five treatments in any period.

The ATTD of DM (*p* < 0.01), GE (*p* < 0.01), EE (*p* < 0.01), OM (*p* < 0.01) and ADF (*p* = 0.02) significantly decreased for pigs fed diets with FVS on d 28, and the nutrient digestibility decreased as the inclusion level of FVS increased (Table 4). However, there was no difference in the ATTD of CP and NDF at d 28. The ATTD of DM, OM, GE and ADF were significantly (linear, *p* < 0.01; quadratic, *p* < 0.05) affected by the inclusion level of FVS. The ATTD of CP and EE linearly decreased (*p* < 0.01) with the increased FVS inclusion level.

Compared with the control diet, the contents of acetate, formate and total SCFAs in feces of piglets fed the FVS diets significantly increased (*p* < 0.05) on d 28. Moreover, the concentration of acetate on d 14 and the concentration of acetate, propionate, butyrate and total SCFAs on d 28 quadratically changed (*p* < 0.05) as the inclusion level of FVS increased (Table 5).

For the biochemical profile in serum, the interleukin-2 (IL-2) level significantly increased for piglets fed diets supplied with FVS compared to those fed the control diet (*p* = 0.03), and the IL-2 content also linearly increased (*p* = 0.02) with the increased inclusion level of FVS (Table 6).

### 3.2. Exp.2

There was no difference in ADG, ADFI, G/F, diarrhea rate and mortality ratio during phase 1 (7–11 kg), phase 2 (11–25 kg), phase 3 (25–50 kg) and the overall period of the animal trial (Table 7). These results were consistent with those from Exp.1, which showed that diet supplemented with 3% or lower levels of FVS did not affect the growth performance of piglets.

On d 14, the ATTD of DM, OM, GE, CP, EE, NDF and ADF were not influenced (*p* > 0.1) by dietary FVS supplementation (Table 8). However, the ATTD of NDF and ADF were significantly decreased for pigs fed diets supplemented with 3% FVS compared with those fed the control and 1.5% FVS diets on d 35. Moreover, pigs fed the FVS diets revealed a significant decrease (*p* < 0.05) in the ATTD of DM, OM and GE on d 35 and d 56, which agreed with the results in Exp.1.

For the SCFAs production, diet with FVS supplementation increased (*p* = 0.02) the contents of lactate and formate in the feces of piglets compared with the control diet on d 14 (Table 9). The concentration of acetate, propionate, formate, butyrate and total SCFAs on d 35 (*p* < 0.05) and the concentration of acetate, propionate, isobutyrate and total SCFAs on d 56 produced in piglets also increased (*p* < 0.05) with the supplementation of dietary FVS compared with the control group.

For the serumal indices, the content of IL-2 on d 14 were higher (*p* < 0.01) for pigs fed the 3% FVS diet than those fed the control diet and 1.5% FVS diet (Table 10), which was consistent with the results of Exp.1. Compared with pigs fed the control diet, an increase (*p* = 0.03) in GH content in serum of piglets fed the 3% FVS diet was observed during phase 1. Compared with the control diet, pigs supplemented with dietary FVS revealed a significantly decrease (*p* < 0.05) in serumal LDL content. Dietary FVS supplementation not only elevated (*p* < 0.05) the values of SOD and GSH-Px in phase 2, but also increased (*p* < 0.01) the activities of GSH-Px in phase 3.

## 4. Discussion

### 4.1. Growth Performance

The results in Exp.1 revealed that high levels of dietary fiber had a negative effect on nutrient digestibility, which were consistent with the study of Wenk [3].

In Exp.1, there were 24% increase in NDF level (from 10.16% to 12.58%) and 12% increase in ADF level (from 3.53% to 3.95%) in diets with 2.5% FVS supplementation compared to the control diet when fed to piglets in the first two weeks, and 26% increase in NDF level (from 10.27% to 12.95%) and 24% increase in ADF level (from 3.78% to 4.69%) in diets with 10% FVS supplementation compared to the control diet when fed to piglets in the last two weeks (Table 2), and we found these changes had little influence on the growth performance of the piglets. However, when the dietary NDF level increased more than 25% (from 10.16% to 12.67%, d 1–14, diets with 5% FVS compared to the control diet), it did affect the growth performance of the piglets (Table 4). Many previous studies have been conducted to evaluate the effects of dietary fiber sources on growth performance of pigs, but few researches focused on the dietary NDF levels. Mateos et al. [23] indicated that young pigs at 6–12 kg liveweight required at least 5–6% NDF in diet for normal growth. British Society of Animal Science [24] recommends 7–13% NDF inclusion in diets for weaned pigs at 10–30 kg liveweight. Our results also indicated the positive effects of NDF inclusion on diet formulation of piglets.

The dietary fiber in swine diet is considered an important factor that could affect the palatability of the diet and the feed intake of pigs [25]. From the results of Exp.1, obviously, high levels of FVS (greater than 5%) in diets affected the palatability and ADFI, which is consistent with the results of most previous studies that have shown the negative effects of high levels of dietary fiber inclusion on ADFI and growth performance of piglets [26,27]. To find out the optimal inclusion level of FVS in piglets’ diets, Exp.2 was conducted with lower inclusion levels of FVS and longer adaption time in the animal trial.

The results on growth performance and ATTD of nutrients in Exp.2 were consistent with those in Exp.1. However, not like the diets used in Exp.1, the diets used in Exp.2 were isoenergetic, indicating that the decreased ATTD of nutrients in Exp.2 with the dietary FVS supplementation were not caused by the decreased DE values among the treatment diets.

In the current study, the growth performance in the two animal trials conducted on piglets were different from those results of the previous studies carried out on broiler chickens. The different digestive physiology between poultry and swine may explain these differences [3]. Regarding to the digestive capacity of pigs for different dietary fiber components, lignin is undigested, both hemicelluloses and cellulose are partly digested with hemicelluloses being more digested than cellulose, and pectins are almost totally digested [28]. Valencia et al. [29] indicated that experimental diets supplemented with 1.25% lignin did not affect the growth performance of pigs weaned at 21 days of age. The FVS contains higher levels of NDF (34.06%), ADF (18.41%) and lignin (3.53%) (Table 1), and obviously, the experimental diets supplemented with greater levels of FVS negatively affected the growth performance of piglets. FVS possesses very complex compositions, resulting in the different effects of FVS inclusion on the digestion and absorption of nutrients in piglets.

### 4.2. Fecal Short Chain Fatty Acids Production and Serumal Indices

In both Exp.1 and Exp.2, we found that dietary FVS supplementation at different levels play a positive role in improving the SCFAs contents for piglets in any phase. Zeng et al. [30] and Wang et al. [7] reported that dietary FVS supplementation increased the SCFAs production in the intestine of broilers, which was consistent with our study. We observed that the production of lactate was markedly different between the two experiments. Given the similarity in weaning day (d 28), diets and environment, we deduced that the difference may be due to the initial body weight (IBW) imparity. In the opinion of Cheng et al. [31], maternal diet during pregnancy modifies microbiota and intestinal development of offspring in a long-term manner. In that way, different IBW may be closely associated with the microbiota and sow diet. Burnett et al. [32] showed that lactic acid producing bacteria were chiefly *Lactobacillus bifidus*. We may consider that it is highly likely that increased IBW led to the decreased lactate-generated microbiota such as *Lactobacillus bifidus*.

Interleukin-2 is secreted by the Th1 cells, which could induce B lymphocyte (B cell), T lymphocyte and natural killer cell (NK) activation and proliferation [33,34]. Dietary FVS supplementation significantly increased the content of IL-2 on d 28 in Exp.1 and on d 14 in Exp.2, which agreed with the results of Mahfuz et al. [9], who showed the content of IL-2 strikingly improved for broilers fed the FVS diets. Therefore, dietary FVS supplementation might play a positive role in enhancing the T and B lymphocyte immune functions in piglets.

Growth hormone (GH) is a form of peptide that can stimulate growth, cell regeneration and reproduction in animals. Our result revealed that FVS supplementation could promote piglets’ growth to some extent during first 2 weeks in Exp.2.

Low density lipoprotein has aroused particular attention among the known risk factors for cardiovascular disease. Previous studies have showed that a reduction in concentrations of LDL cholesterol could markedly reduce the risk of stroke and coronary heart disease incidence and mortality [35]. Many previous studies have also shown that mushroom has excellent effects on lowering the LDL content [36,37]. Therefore, dietary FVS could benefit the health of piglets through lowering the LDL content in serum.

Results of antioxidant capacity showed that lipid peroxidation could be decreased by dietary FVS supplementation. It is well known that endogenous antioxidant enzymes play crucial roles in preventing oxidative damage in the body of the host [38]. In the organism, there are many different endogenous enzymatic antioxidant defenses, either in intracellular or extracellular medium, such as SOD, CAT and GSH-Px. SOD converts O_2_^−^ into H_2_O_2_, which is then detoxified to water either by GSH-Px in the mitochondria or by CAT in the peroxysomes, cytosol or nucleus [38]. GSH-Px are a group of selenoenzymes in which selenium is essential on their biosynthesis [39]. The dietary selenium supplementation is crucial for antioxidant enzyme defenses, and mushrooms have been generally found to contain selenium in good quantity [40]. These results suggested that FVS could act as a potential source of natural antioxidants to improve the antioxidant capacity of piglets.

*Flammulina velutipes* serves as an excellent fibrous source that exhibit effects of cholesterol-lowering [41], antioxidant, immunomodulatory, anti-inflammatory and anti-tumor. Previous studies indicated that the polysaccharides, oligosaccharides and extracts prepared from fruiting FV body showed strong antioxidant activities [42,43,44]. For instance, the *Flammulina velutipes* polysaccharide (FVP) was reported to have potential effects in increasing the activities of SOD, CAT and GSH-Px, and in reducing the contents of MDA in kidney of humans [45]. The FVP was also reported to be able to increase the proliferation of macrophage [46], promote the secretion of interleukin-1β, interleukin-6 and tumor necrosis factor-α secreted by macrophages and stimulate the lymphocyte proliferation [43]. Moreover, FIP-fve, a fungal immunomodulatory protein (FIP) isolated from FV, was reported to exhibit some biological activities such as anti-allergy, anti-tumor, immunomodulation [47,48] and anti-inflammatory function [49,50].

In summary, dietary FVS supplementation has been shown to have positive effects on improving immune status, SCFAs production, GH content in serum, antioxidant ability, and decreasing the LDL content in serum of piglets. There are many bioactive compounds reported in FV, however, it is still unknown which compound plays the most important role in promoting the health of the host. More future studies can be done on the beneficial effects of FV and its by-products.

## 5. Conclusions

In conclusion, piglets fed diets with 3% or lower inclusion levels of FVS showed no difference on the growth performance compared with those fed the control diet during d 1–28 and d 1–56. Dietary FVS supplementation obviously decreased the ATTD of some nutrients on d 28, d 35 and d 56, but no significant change on d 14. Although piglets fed diets with higher levels of FVS showed impaired growth performance and nutrient digestibility, dietary FVS supplementation improved the SCFA production in feces, the antioxidant ability, the IL-2 and GH contents in serum, and reduced the LDL content in serum.

Overall, FVS is a promising alternative fibrous ingredient in diets fed to piglets. The results of this study provided a possible route for the utilization of the FV by-products, and maybe also for the other by-products in the edible fungi industry. Effective utilization of mushroom by-products can avoid the waste of resources and has direct positive effects on economic development and environmental pollution reduction.

## Figures and Tables

**Table 1:** Analyzed chemical compositions of *Flammulina velutipes* stem waste (FVS) (%, as-fed basis, unless otherwise indicated) ^1^.

Item	FVS
Dry matter	89.70
Gross energy, MJ/kg	15.88
Crude protein	13.69
Ether extract	1.60
Ash	8.60
Crude fiber	15.30
Neutral detergent fiber	34.06
Acid detergent fiber	18.41
Lignin	3.53
Calcium	0.58
Total phosphorus	0.45
Essential amino acids	
Lysine	0.57
Methionine	0.14
Threonine	0.47
Tryptophan	0.15
Valine	0.46
Leucine	0.61
Isoleucine	0.44
Phenylalanine	0.47
Histidine	0.23
Arginine	0.42
Nonessential amino acids	
Tyrosine	0.39
Serine	0.43
Glutamic acid	1.61
Proline	0.49
Glycine	0.45
Alanine	0.71
Cysteine	0.13
Aspartic acid	0.84
Total essential amino acids	3.96
Total nonessential amino acids	5.05
Total amino acids	9.01

^1^ All values are the results of analysis conducted in duplicate.

**Table 2:** Ingredients and analyzed nutrient levels of the experimental diets used in Exp.1 (%, as-fed basis).

Item	d 1 to 14					d 15 to 28				
	Control	FVS				Control	FVS			
		2.5%	5%	7.5%	10%		2.5%	5%	7.5%	10%
Ingredients										
Corn	59.94	58.21	56.85	55.71	54.41	61.71	60.52	59.22	57.97	56.80
Soybean meal	15.00	14.30	13.16	11.80	10.60	15.00	13.80	12.60	11.35	10.02
Fish meal	5.00	5.00	5.00	5.00	5.00	5.00	5.00	5.00	5.00	5.00
Whey powder	8.00	8.00	8.00	8.00	8.00	8.00	8.00	8.00	8.00	8.00
Soy protein concentrate	6.00	6.00	6.00	6.00	6.00	6.00	6.00	6.00	6.00	6.00
FVS ^1^	-	2.50	5.00	7.50	10.00	0.00	2.50	5.00	7.50	10.00
Soybean oil	2.75	2.70	2.70	2.70	2.70	1.40	1.30	1.30	1.30	1.30
Choline chloride	0.20	0.20	0.20	0.20	0.20	0.20	0.20	0.20	0.20	0.20
Dicalcium phosphate	1.00	1.00	1.00	1.00	1.00	0.60	0.60	0.60	0.60	0.60
Limestone	0.70	0.70	0.70	0.70	0.70	0.67	0.67	0.67	0.67	0.67
Sodium chloride	0.20	0.20	0.20	0.20	0.20	0.20	0.20	0.20	0.20	0.20
L-lysine·HCl	0.45	0.44	0.44	0.44	0.44	0.29	0.28	0.28	0.28	0.28
DL-Methionine	0.08	0.08	0.08	0.08	0.08	0.04	0.04	0.04	0.04	0.04
L-Threonine	0.15	0.14	0.14	0.14	0.14	0.08	0.08	0.08	0.08	0.08
L-Tryptophan	0.03	0.03	0.03	0.03	0.03	0.01	0.01	0.01	0.01	0.01
Chromic oxide	-	-	-	-	-	0.30	0.30	0.30	0.30	0.30
Vitamin mineral premix ^2^	0.50	0.50	0.50	0.50	0.50	0.50	0.50	0.50	0.50	0.50
Analyzed nutrient level										
Dry matter	89.95	90.38	90.45	90.51	90.73	91.01	91.07	91.16	91.06	90.76
Crude protein	19.16	19.40	18.92	18.49	18.15	18.33	17.90	18.68	17.73	17.14
Gross energy, MJ/kg	16.88	16.82	16.88	16.80	16.84	16.78	16.67	16.72	16.61	16.42
Ash	4.95	5.04	5.17	5.51	5.56	4.94	5.09	5.31	5.44	5.50
Ether extract	5.61	6.35	6.13	5.80	6.07	5.43	5.48	4.83	5.15	4.81
Neutral detergent fiber	10.16	12.58	12.67	12.69	13.57	10.27	12.46	12.63	12.77	12.95
Acid detergent fiber	3.53	3.95	4.33	4.31	4.60	3.78	4.12	4.26	4.31	4.69
Calculated nutrient level										
DE, MJ/kg	14.83	14.55	14.29	14.02	13.75	14.59	14.30	14.03	13.77	13.50
SID Lys	1.35	1.33	1.30	1.26	1.23	1.23	1.19	1.16	1.13	1.10
SID Met	0.40	0.39	0.39	0.38	0.37	0.36	0.35	0.35	0.34	0.33
SID Thr	0.79	0.77	0.75	0.74	0.72	0.73	0.71	0.69	0.68	0.66
SID Trp	0.22	0.22	0.21	0.21	0.20	0.20	0.20	0.19	0.19	0.18
DE/CP	0.75	0.74	0.74	0.73	0.73	0.74	0.74	0.73	0.73	0.73
SID Lys/DE	0.09	0.09	0.09	0.09	0.09	0.08	0.08	0.08	0.08	0.08
SID Lys/SID Met	3.39	3.37	3.36	3.34	3.32	3.40	3.36	3.35	3.33	3.31
SID Lys/SID Thr	1.71	1.72	1.72	1.72	1.72	1.69	1.68	1.68	1.68	1.67
SID Lys/SID Trp	6.09	6.05	6.06	6.08	6.09	6.06	6.03	6.04	6.05	6.06

^1^ FVS: *Flammulina velutipes* stem waste. ^2^ Vitamin mineral premix provided the following quantities of vitamins and minerals per kg of diet: vitamin A, 12,000 IU; vitamin D_3_, 3000 IU; vitamin E, 30 IU; vitamin K_3_, 2.5 mg; vitamin B_12_,20.0 µg; riboflavin, 4.0 mg; pantothenic acid, 12.5 mg; niacin, 40 mg; choline chloride, 400 mg; folacin, 0.7 mg; thiamine 2.5 mg; pyridoxine 3.0 mg; biotin, 70 µg; Mn, 30 mg (MnO); Fe, 100 mg (FeSO_4_·H_2_O); Zn, 80 mg (ZnO); Cu, 90 mg (CuSO_4_·5H_2_O); I, 0.25 mg (KI); Se, 0.15 mg (Na_2_SeO_3_). CP, crude protein; DE, digestible energy; SID, standardized ileal digestible; Lys, lysine; Thr, threonine; Met, methionine; Trp, tryptophan.

**Table 3:** Ingredients and analyzed nutrient levels of the experimental diets used in Exp.2 (%, as-fed basis).

Item	Phase 1: d 1 to 14			Phase 2: d 15 to 35			Phase 3: d 36 to 56		
	Control	FVS		Control	FVS		Control	FVS	
		1.5%	3%		1.5%	3%		1.5%	3%
Ingredients									
Corn	59.50	57.30	55.08	61.66	59.42	57.21	69.84	67.64	65.43
Soybean meal	15.00	15.00	15.00	15.00	15.00	15.00	26.00	26.00	26.00
Fish meal	5.00	5.00	5.00	5.00	5.00	5.00	-	-	-
Whey powder	8.00	8.00	8.00	8.00	8.00	8.00	-	-	-
Soy protein concentrate	6.00	6.00	6.00	6.00	6.00	6.00	-	-	-
FVS ^1^	-	1.50	3.00	-	1.50	3.00	-	1.50	3.00
Soybean oil	2.90	3.60	4.30	1.45	2.18	2.88	0.58	1.28	1.98
Choline chloride	0.20	0.20	0.20	0.20	0.20	0.20	-	-	-
Dicalcium phosphate	1.00	1.00	1.00	0.60	0.60	0.60	1.26	1.26	1.26
Limestone	0.70	0.70	0.70	0.67	0.67	0.67	0.82	0.82	0.82
Sodium chloride	0.20	0.20	0.20	0.20	0.20	0.20	0.35	0.35	0.35
L-lysine·HCl	0.45	0.45	0.45	0.29	0.29	0.30	0.25	0.25	0.25
DL-Methionine	0.07	0.07	0.08	0.04	0.04	0.04	0.03	0.03	0.03
L-Threonine	0.15	0.15	0.16	0.08	0.09	0.09	0.07	0.07	0.08
L-Tryptophan	0.03	0.03	0.03	0.01	0.01	0.01	-	-	-
Chromic oxide	0.30	0.30	0.30	0.30	0.30	0.30	0.30	0.30	0.30
Vitamin mineral premix ^2^	0.50	0.50	0.50	0.50	0.50	0.50	0.50	0.50	0.50
Analyzed nutrient level									
Dry matter	89.68	89.61	89.62	88.82	88.99	89.08	86.40	86.67	87.58
Crude protein	20.33	19.35	19.04	17.42	20.00	18.89	17.06	15.23	16.84
Gross energy, MJ/kg	16.91	17.10	17.18	16.69	16.78	16.96	15.92	16.17	16.51
Ash	5.53	5.34	5.54	5.08	5.14	4.99	4.64	4.59	5.05
Ether extract	5.90	6.55	5.77	4.10	4.73	4.53	2.81	3.67	3.56
Neutral detergent fiber	10.85	11.24	11.29	11.20	12.49	14.33	10.13	11.26	12.97
Acid detergent fiber	4.64	4.64	4.68	4.21	4.24	5.09	4.00	4.04	5.26
Calculated nutrient level									
DE, MJ/kg	14.82	14.82	14.82	14.60	14.60	14.60	14.23	14.23	14.23
SID Lys	1.35	1.35	1.35	1.23	1.23	1.23	0.98	0.98	0.98
SID Met	0.39	0.39	0.39	0.36	0.36	0.36	0.28	0.28	0.28
SID Thr	0.79	0.79	0.79	0.73	0.73	0.73	0.60	0.60	0.60
SID Trp	0.22	0.22	0.22	0.20	0.20	0.20	0.17	0.17	0.17
SID Lys/DE	0.09	0.09	0.09	0.08	0.08	0.08	0.07	0.07	0.07
SID Lys/SID Met	3.48	3.50	3.42	3.40	3.41	3.45	3.49	3.51	3.53
SID Lys/SID Thr	1.71	1.71	1.70	1.69	1.68	1.69	1.64	1.64	1.62
SID Lys/SID Trp	6.10	6.09	6.08	6.06	6.05	6.07	5.73	5.72	5.70

^1^ FVS: *Flammulina velutipes* stem waste. ^2^ Vitamin mineral premix provided the following quantities of vitamins and minerals per kg of diet: vitamin A, 12,000 IU; vitamin D3, 3000 IU; vitamin E, 30 IU; vitamin K3, 2.5 mg; vitamin B12, 20.0 µg; riboflavin, 4.0 mg; pantothenic acid, 12.5 mg; niacin, 40 mg; choline chloride, 400 mg; folacin, 0.7 mg; thiamine 2.5 mg; pyridoxine 3.0 mg; biotin, 70 µg; Mn, 30 mg (MnO); Fe, 100 mg (FeSO4·H2O); Zn, 80 mg (ZnO); Cu, 90 mg (CuSO4·5H2O); I, 0.25 mg (KI); Se, 0.15 mg (Na2SeO3). DE, digestible energy; SID, standardized ileal digestible; Lys, lysine; Thr, threonine; Met, methionine; Trp, tryptophan.

**Table 4:** Effects of *Flammulina velutipes* stem waste (FVS) supplementation in diets fed to weaned piglets on growth performance and apparent total tract digestibility (ATTD) of nutrients during day 1–28 (Exp.1).

Item	Control	FVS				SEM	*p*-Value		
		2.5%	5%	7.5%	10%		ANOVA	Linear	Quadratic
**BW d 0, kg**	**6.88**	**6.89**	**6.90**	**6.90**	**6.90**	0.01	0.65	0.41	0.63
d 1 to 14									
ADG, g	382.2 ^a^	372.7 ^a^	318.3 ^b^	319.7 ^b^	293.8 ^b^	15.0	<0.01	<0.01	0.68
ADFI, g	533.1 ^a^	501.5 ^a^	457.7 ^bc^	455.3 ^bc^	436.3 ^c^	14.6	<0.01	<0.01	0.24
G/F	0.71	0.74	0.69	0.70	0.67	0.02	0.12	0.05	0.41
Diarrhea rate, %	4.05	3.81	3.57	1.91	3.10	1.12	0.69	0.29	0.73
Mortality ratio, %	6.67	0	3.33	3.33	6.67	-	0.95	0.35	0.12
d 15 to 28									
ADG, g	431.0	451.8	408.9	364.9	347.9	27.0	0.07	<0.01	0.44
ADFI, g	820.6	891.3	810.2	784.1	794.2	39.1	0.36	0.19	0.64
G/F	0.53	0.50	0.50	0.47	0.44	0.02	0.08	<0.01	0.58
Diarrhea rate, %	0.95	0.48	0.00	0.48	0.48	0.47	0.73	0.52	0.28
Mortality ratio, %	3.57 ^a^	0 ^b^	0 ^b^	3.44 ^a^	0 ^b^	-	<0.01	-	1.00
d 1–28									
ADG, g	406.6 ^a^	411.7 ^a^	366.3 ^ab^	344.0 ^b^	325.0 ^b^	15.0	<0.01	<0.01	0.66
ADFI, g	676.9	699.0	636.3	630.0	658.4	22.8	0.22	0.16	0.43
G/F	0.60 ^a^	0.59 ^a^	0.58 ^a^	0.55 ^ab^	0.50 ^b^	0.02	0.01	<0.01	0.24
Diarrhea rate, %	2.50	2.15	1.79	1.91	1.79	0.62	0.66	0.24	0.48
Mortality ratio, %	10.00	0	3.33	6.67	6.67	-	0.91	0.35	0.09
ATTD of nutrients, % (d 28)									
Dry matter	88.45 ^a^	82.50 ^b^	81.75 ^bc^	80.48 ^c^	80.55 ^c^	0.50	<0.01	<0.01	0.01
Organic matter	88.13 ^a^	85.70 ^b^	84.80 ^bc^	83.77 ^c^	83.80 ^c^	0.45	<0.01	<0.01	0.01
Gross energy	85.28 ^a^	82.29 ^b^	81.26 ^bc^	79.93 ^c^	79.75 ^c^	0.55	<0.01	<0.01	0.03
Crude protein	79.87	75.66	76.22	74.12	73.55	1.50	0.06	<0.01	0.41
Ether extract	74.72 ^a^	71.87 ^ab^	66.14 ^c^	68.28 ^bc^	65.94 ^c^	1.23	<0.01	<0.01	0.08
Neutral detergent fiber	52.90	52.29	51.62	47.45	51.06	1.72	0.23	0.12	0.44
Acid detergent fiber	48.94 ^a^	39.72 ^ab^	42.04 ^ab^	36.28 ^b^	40.38 ^ab^	2.35	0.02	<0.01	0.04

^a–c^ Least squares means within different superscripts differ (*p* < 0.05). n = 6. BW, body weight; ADG, average daily gain; ADFI, average daily feed intake; G/F, gain to feed ratio; SEM, standard error of the mean; ANOVA, analysis of variance.

**Table 5:** Effects of dietary *Flammulina velutipes* stem waste (FVS) supplementation on short chain fatty acids (SCFAs) production measured in fresh feces of weaned piglets on day 14 and day 28 (mg/g) (Exp.1).

Item	Control	FVS				SEM	*p*-Value		
		2.5%	5%	7.5%	10%		ANOVA	Linear	Quadratic
**d 14**									
Lactate	2.45	2.03	3.45	1.75	1.96	0.44	0.08	0.36	0.26
Acetate	4.39	5.44	5.67	4.92	4.99	0.39	0.22	0.56	0.04
Propionate	1.65	2.14	2.00	1.90	1.88	0.23	0.67	0.74	0.24
Formate	0.15	0.18	0.18	0.18	0.16	0.02	0.70	0.99	0.63
Isobutyrate	0.11	0.16	0.09	0.13	0.10	0.02	0.26	0.60	0.60
Butyrate	0.98	1.62	1.02	0.91	0.91	0.18	0.05	0.16	0.27
Isovalerate	0.43	0.36	0.35	0.36	0.28	0.07	0.67	0.24	0.97
Valerate	0.24	0.37	0.25	0.20	0.15	0.06	0.17	0.09	0.27
Total	10.40	12.31	13.00	10.34	10.43	0.94	0.17	0.51	0.05
**d 28**									
Lactate	1.11	1.67	1.97	1.47	1.73	0.50	0.80	0.53	0.48
Acetate	3.75 ^b^	5.71 ^a^	4.98 ^ab^	5.44 ^a^	4.75 ^ab^	0.40	0.02	0.19	0.01
Propionate	1.57	2.25	2.04	2.02	1.88	0.15	0.07	0.45	0.02
Formate	0.17 ^b^	0.26 ^a^	0.24 ^a^	0.25 ^a^	0.24 ^a^	0.02	0.01	0.12	0.13
Isobutyrate	0.14	0.14	0.12	0.10	0.12	0.02	0.64	0.22	0.76
Butyrate	0.88	1.26	1.27	1.16	1.08	0.12	0.16	0.44	0.03
Isovalerate	0.25	0.25	0.22	0.23	0.21	0.03	0.74	0.23	0.95
Valerate	0.27	0.31	0.23	0.23	0.26	0.05	0.76	0.50	0.70
Total	8.15 ^b^	11.85 ^a^	11.07 ^ab^	10.90 ^ab^	10.27 ^ab^	0.81	0.04	0.22	0.02

^a,b^ Least squares means within different superscripts differ (*p* < 0.05). n = 6. SEM, standard error of the mean; ANOVA, analysis of variance.

**Table 6:** Effects of dietary *Flammulina velutipes* stem waste (FVS) supplementation on serum profile of weaned piglets on day 28 (Exp.1).

Item	Control	FVS				SEM	*p*-Value		
		2.5%	5%	7.5%	10%		ANOVA	Linear	Quadratic
IgA (g/L)	1.09	1.15	1.16	1.21	1.19	0.05	0.46	0.28	0.68
IgG (g/L)	19.96	20.70	20.29	20.02	20.10	0.64	0.93	0.87	0.68
IgM (g/L)	2.39	2.41	2.43	2.40	2.39	0.04	0.95	0.93	0.61
GSH-Px (U/mL)	77.29	87.86	88.68	83.48	70.69	6.24	0.30	0.45	0.06
CAT (U/mL)	53.34	56.41	55.40	59.27	47.91	4.13	0.40	0.58	0.17
T-AOC (U/mL)	10.61	10.52	10.78	10.28	9.34	0.34	0.06	0.05	0.14
MDA (nmol/mL)	4.07	3.92	3.55	3.63	4.67	0.30	0.10	0.38	0.03
IL-2 (pg/mL)	21.78 ^b^	23.77 ^ab^	25.96 ^ab^	23.22 ^ab^	32.03 ^a^	2.24	0.03	0.02	0.38
IL-6 (pg/mL)	124.81	128.76	137.69	129.62	145.37	6.08	0.16	0.08	0.80
HDL(mmol/L)	0.33	0.35	0.34	0.28	0.27	0.04	0.50	0.18	0.52
LDL (mmol/L)	0.47	0.54	0.56	0.43	0.47	0.08	0.74	0.68	0.55
TC (mmol/L)	1.00	1.11	1.14	0.77	0.84	0.14	0.32	0.21	0.42
TG (mmol/L)	0.35	0.40	0.35	0.32	0.39	0.04	0.73	0.99	0.81
ALB (g/L)	11.23	13.36	11.15	10.37	10.66	0.94	0.22	0.22	0.57
TP (g/L)	32.36	34.38	29.88	32.01	26.58	2.55	0.29	0.14	0.46
Urea(mmol/L)	3.12	3.43	3.44	2.87	3.34	0.18	0.18	0.85	0.72
GLU(mmol/L)	2.93	3.42	3.22	2.74	2.80	0.27	0.36	0.30	0.28

^a,b^ Least squares means within a row with different superscripts differ (*p* < 0.05). n = 6. IgA, immunoglobulin A; IgG, Immunoglobulin G; IgM, immunoglobulin M; GSH-Px, glutathione peroxidase; CAT, catalase; T-AOC, total antioxidant capacity; MDA, malondialdehyde; IL-2, interleukin-2; IL-6, interleukin-6; HDL, high density lipoprotein; LDL, low density lipoprotein; TC, total cholesterol; TG, triglyceride; ALB, albumin; TP, total protein; GLU, glucose; SEM, standard error of the mean; ANOVA, analysis of variance.

**Table 7:** Effects of *Flammulina velutipes* stem waste (FVS) supplementation in diets fed to weaned piglets on growth performance during day 1–56 (Exp.2).

Item	Control	FVS		SEM	*p*-Value
		1.5%	3%		
BW d 0, kg	8.20	8.22	8.19	0.04	0.91
Phase 1 (7–11 kg)					
ADG, g	229.8	274.5	242.2	31.0	0.60
ADFI, g	372.9	409.6	413.0	33.6	0.66
G/F	0.61	0.66	0.59	0.05	0.60
Diarrhea rate, %	4.46	3.87	3.57	0.53	0.52
Mortality ratio, %	8.33	0	4.17	-	0.84
Phase 2 (11–25 kg)					
ADG, g	482.2	484.0	490.2	12.6	0.90
ADFI, g	859.4	869.3	864.3	43.9	0.99
G/F	0.56	0.56	0.58	0.02	0.77
Diarrhea rate, %	1.88	1.25	1.87	0.36	0.42
Mortality ratio, %	0	0	0	-	1.00
Phase 3 (25–50 kg)					
ADG, g	669.8	625.9	615.4	16.8	0.13
ADFI, g	1361.9	1418.2	1490.9	90.5	0.62
G/F	0.49	0.44	0.42	0.02	0.14
Diarrhea rate, %	0.76	0.37	0.38	0.22	0.42
Mortality ratio, %	0	0	0	-	1.00
Overall period					
ADG, g	492.8	487.4	478.9	16.8	0.84
ADFI, g	935.2	970.0	998.8	53.6	0.72
G/F	0.53	0.50	0.49	0.02	0.44
Diarrhea rate, %	2.08	1.56	1.71	0.25	0.38
Mortality ratio, %	8.33	0	4.17	-	0.84

n = 6. BW, body weight; ADG, average daily gain; ADFI, average daily feed intake; G/F, gain to feed ratio; ATTD, apparent total tract digestibility; SEM, standard error of the mean.

**Table 8:** Effects of *Flammulina velutipes* stem waste (FVS) supplementation in diets fed to weaned piglets on apparent total tract digestibility (ATTD) of nutrients during day 1–56 (%, Exp.2).

Item	Control	FVS		SEM	*p*-Value
		1.5%	3%		
Phase 1 (7–11 kg, d 14)					
Dry matter	80.78	81.41	79.43	0.70	0.21
Organic matter	84.15	84.68	82.81	0.63	0.18
Gross energy	79.81	80.65	78.84	0.99	0.48
Crude protein	75.55	76.24	71.66	1.38	0.11
Ether extract	54.86	62.18	60.02	4.20	0.49
Neutral detergent fiber	42.71	39.75	37.65	4.09	0.69
Acid detergent fiber	35.82	30.16	29.55	4.36	0.57
Phase 2 (11–25 kg, d 35)					
Dry matter	84.81 ^a^	83.08 ^b^	82.42 ^b^	0.36	0.01
Organic matter	87.52 ^a^	86.00 ^b^	85.26 ^b^	0.34	0.01
Gross energy	84.78 ^a^	83.01 ^b^	82.36 ^b^	0.48	0.03
Crude protein	77.82	76.80	77.34	0.77	0.66
Ether extract	65.85	67.51	64.96	1.22	0.38
Neutral detergent fiber	59.25 ^a^	60.56 ^a^	50.82 ^b^	2.06	0.03
Acid detergent fiber	59.25 ^a^	60.56 ^a^	50.82 ^b^	2.41	0.02
Phase 3 (25–50 kg, d 56)					
Dry matter	86.45 ^a^	84.34 ^b^	84.24 ^b^	0.41	0.01
Organic matter	89.24 ^a^	87.55 ^b^	87.27 ^b^	0.34	0.01
Gross energy	86.28 ^a^	84.11 ^b^	84.17 ^b^	0.47	0.03
Crude protein	82.18	78.03	80.75	1.31	0.15
Ether extract	58.22	58.76	57.07	2.72	0.91
Neutral detergent fiber	49.92	52.36	55.91	4.25	0.63
Acid detergent fiber	50.52	47.95	57.13	3.75	0.28

^a,b^ Least squares means within different superscripts differ (*p* < 0.05). n = 6. SEM, standard error of the mean.

**Table 9:** Effects of dietary *Flammulina velutipes* stem waste (FVS) supplementation on short chain fatty acids (SCFAs) production measured in fresh feces of piglets on day 14, day 28 and day 56 (mg/g) (Exp.2).

Item	Control	FVS		SEM	*p*-Value
		1.5%	3%		
Phase 1 (d 14)					
Lactate	0.07 ^b^	0.18 ^a^	0.19 ^a^	0.02	0.02
Acetate	3.75	3.34	4.44	0.42	0.25
Propionate	2.72	2.55	3.34	0.35	0.30
Formate	0.01 ^b^	0.12 ^a^	0.10 ^a^	0.02	0.02
Isobutyrate	0.10	0.48	0.38	0.09	0.06
Butyrate	0.69	1.04	1.07	0.13	0.14
Isovalerate	0.33	0.55	0.24	0.23	0.65
Valerate	0.27	0.09	0.33	0.10	0.27
Total	7.93	8.35	10.10	0.82	0.22
Phase 2 (d 35)					
Lactate	0.02	0.05	0.03	0.02	0.52
Acetate	3.31 ^b^	4.66 ^a^	5.38 ^a^	0.25	<0.01
Propionate	2.25 ^b^	3.02 ^ab^	3.65 ^a^	0.28	0.03
Formate	0.05 ^c^	0.12 ^a^	0.08 ^b^	0.01	<0.01
Isobutyrate	1.79	2.59	3.43	0.43	0.09
Butyrate	0.12 ^b^	0.33 ^a^	0.17 ^ab^	0.05	<0.05
Isovalerate	0.18	0.42	0.34	0.07	0.12
Valerate	0.38	0.34	0.69	0.11	0.12
Total	8.11 ^b^	11.54 ^ab^	13.79 ^a^	1.02	0.02
Phase 3 (d 56)					
Lactate	0.01	0.06	0.03	0.01	0.14
Acetate	3.52 ^b^	4.10 ^b^	5.38 ^a^	0.33	0.02
Propionate	1.74 ^b^	2.88 ^ab^	3.96 ^a^	0.46	0.04
Formate	0.20	0.09	0.10	0.08	0.59
Isobutyrate	1.59 ^b^	1.96 ^b^	2.98 ^a^	0.21	0.01
Butyrate	0.09	0.15	0.20	0.06	0.43
Isovalerate	0.19	0.18	0.34	0.08	0.35
Valerate	0.59	0.46	0.55	0.07	0.47
Total	7.93 ^b^	9.88 ^b^	13.55 ^a^	10.00	0.02

^a,b^ Least squares means within different superscripts differ (*p* < 0.05). n = 6. SEM, standard error of the mean.

**Table 10:** Effects of dietary *Flammulina velutipes* stem waste (FVS) supplementation on serum profile of piglets on day 14, day 35 and day 56 (Exp.2).

Item	Control	FVS		SEM	*p*-Value
		1.5%	3%		
Phase 1 (7–11 kg, d 14)					
IgA (g/L)	17.56	19.96	25.06	4.87	0.58
IgG (g/L)	7.83	12.81	11.25	2.14	0.34
IgM (g/L)	7.47	10.04	13.00	2.95	0.48
GSH-Px (U/mL)	111.90	116.69	126.85	3.31	0.07
SOD (U/mL)	48.35	48.97	49.51	0.69	0.54
T-AOC (U/mL)	14.00	15.78	13.38	0.52	0.07
MDA (nmol/mL)	1.09	1.18	1.16	0.02	0.07
IL-2 (pg/mL)	29.87 ^b^	34.04 ^b^	63.07 ^a^	2.39	<0.01
HDL (mmol/L)	0.50	0.69	0.75	0.09	0.22
LDL (mmol/L)	1.26	0.95	0.86	0.09	0.08
TC (mmol/L)	2.07	1.97	1.98	0.20	0.92
TG (mmol/L)	0.69	0.71	0.80	0.11	0.78
GH (ng/mL)	1.28 ^b^	1.44 ^b^	2.00 ^a^	0.12	0.03
Phase 2 (11–25 kg, d 35)					
IgA (g/L)	15.86	18.72	20.53	1.44	0.18
IgG (g/L)	8.41	10.58	10.71	0.84	0.21
IgM (g/L)	9.09	10.11	10.39	0.89	0.59
GSH-Px (U/mL)	117.97 ^b^	138.92 ^a^	141.56 ^a^	3.98	0.02
SOD (U/mL)	46.76 ^b^	48.81 ^a^	49.89 ^a^	0.39	0.01
T-AOC (U/mL)	11.85	13.64	11.86	0.42	0.06
MDA (nmol/mL)	1.10	1.15	1.11	0.04	0.68
IL-2 (pg/mL)	29.31	52.48	27.54	8.66	0.19
HDL (mmol/L)	0.69	0.85	0.85	0.09	0.46
LDL (mmol/L)	1.13	1.19	0.99	0.06	0.20
TC (mmol/L)	2.15	2.39	2.14	0.08	0.16
TG (mmol/L)	0.73	0.76	0.66	0.03	0.22
GH (ng/mL)	1.39	1.65	1.80	0.26	0.57
Phase 3 (25–50 kg, d 56)					
IgA (g/L)	15.06	18.65	17.59	1.01	0.14
IgG (g/L)	8.47	9.81	9.65	0.60	0.33
IgM (g/L)	8.27	10.14	9.91	0.56	0.14
GSH-Px (U/mL)	134.69 ^b^	182.44 ^a^	182.93 ^a^	4.47	<0.01
SOD (U/mL)	46.91	45.98	45.12	2.05	0.83
T-AOC (U/mL)	13.99	13.61	14.96	0.90	0.59
MDA (nmol/mL)	1.13	1.15	1.14	0.03	0.88
IL-2 (pg/mL)	32.75	35.04	34.95	2.23	0.73
HDL (mmol/L)	0.79	0.79	0.86	0.07	0.73
LDL (mmol/L)	1.35 ^a^	1.20 ^ab^	1.12 ^b^	0.04	<0.05
TC (mmol/L)	2.24	2.46	2.38	0.13	0.54
TG (mmol/L)	0.74	0.72	0.69	0.10	0.95
GH (ng/mL)	1.78	1.68	1.52	0.17	0.58

^a,b^ Least squares means within a row with different superscripts differ (*p* < 0.05). n = 6. IgA, immunoglobulin A; IgG, Immunoglobulin G; IgM, immunoglobulin M; GSH-Px, glutathione peroxidase; SOD, superoxide dismutase; T-AOC, total antioxidant capacity; MDA, malondialdehyde; IL-2, interleukin-2; HDL, high density lipoprotein; LDL, low density lipoprotein; TC, total cholesterol; TG, triglyceride; GH, growth hormone; SEM, standard error of the mean.
